# Micronutrient Patterns and Low Intake of Vitamin A, Vitamin D, Folate, Magnesium, and Potassium Among Prediabetes and Type 2 Diabetes Patients

**DOI:** 10.7759/cureus.60906

**Published:** 2024-05-23

**Authors:** Oana C Iatcu, Andrei Lobiuc, Mihai Covasa

**Affiliations:** 1 Department of Medicine, Faculty of Medicine, University of Medicine and Pharmacy "Grigore T. Popa", Iasi, ROU; 2 Department of Biomedical Sciences, College of Medicine and Biological Sciences, “Stefan cel Mare” University of Suceava, Suceava, ROU

**Keywords:** dietary intake, recommended dietary intake, prediabetes, minerals, vitamins

## Abstract

Background

Assessing micronutrient intake is important in identifying deficiencies that may contribute to insulin resistance, poor glycemic control, and increased risk of diabetes-related complications. The study's objectives were to evaluate micronutrient intake in prediabetes (PD) and type 2 diabetes (T2DM) patients compared to recommended dietary intakes (RDI) and to determine the associations between the micronutrient patterns and both anthropometric measurements and biomarkers of diabetes.

Methods

This cross-sectional study was conducted on 349 patients with T2DM and 252 patients with PD. Micronutrient intake was evaluated using a validated food frequency questionnaire. Micronutrient patterns were extracted from factor analysis using principal component analysis with varimax rotation. Participants in the highest tertile were considered to have the highest adherence to the corresponding micronutrient pattern.

Results

T2DM patients had a significantly lower intake of vitamin E (9.4 ± 0.2 vs. 10.1 ± 0.3 mg; p = 0.048), vitamin D (44.3 ± 1.1 vs. 48.9 ± 1.7 IU; p = 0.020), and thiamin (1.3 ± 0.1 vs. 1.4 ± 0.1 mg; p = 0.013) compared to PD patients. All patients had a significantly lower intake of vitamin A, vitamin D, folate, magnesium, and potassium and a significantly higher intake of vitamin B12 and copper compared to RDI. Three distinct micronutrient patterns were identified within each group. In the PD group, the Fe-Mn-Se pattern correlated significantly with waist circumference (WC) and fasting plasma glucose (FPG). The Vit.C-K-Folate pattern showed significant associations with body fat (BF). The Vit.B2-P-Vit.B12 pattern was significantly linked to WC, body mass index (BMI), BF, FPG, and serum insulin (SI). For the T2DM patients, the K-Folate-Mg pattern displayed an inverse and significant association with weight and WC. The Iron-Se-Vit.B3 pattern showed a significant association with low-density lipoprotein (LDL) cholesterol, triglycerides, and total cholesterol. The Vit.B2-P-Ca pattern was significantly associated with fasting plasma glucose (FPG).

Conclusion

This study demonstrated that T2DM patients had significantly lower vitamin E, vitamin D, and thiamin intake than PD patients. Both T2DM and PD patients had a significantly lower intake of vitamin A, vitamin D, folate, magnesium, and potassium compared to the RDI. Among the identified micronutrient patterns, only the K-Folate-Mg pattern exhibited a significant association with reduced body weight and WC.

## Introduction

Diabetes remains a global health priority in both developed and developing countries with an estimated 643 million adults with diabetes by 2030 and 783 million by 2045 [1]. This rapid increase in cases of diabetes and prediabetes is due to several factors, including but not limited to population aging, urbanization, sedentary lifestyle, and obesity [2]. Thus, improving individuals’ lifestyle that incorporates both nutrition and physical activity and smoking cessation could contribute to reducing the risk of diabetes [3]. Until now, the role of nutrition in the prevention and management of diabetes has been largely focused on the intake of macronutrients and less on the intake of micronutrients, even if micronutrients play a vital role in various physiological processes, including glucose metabolism, insulin sensitivity, and inflammation, which are key factors in the development and management of diabetes [4]. Assessing micronutrient intake becomes significant as it can identify deficiencies or imbalances that may contribute to insulin resistance, poor glycemic control, and increased risk of diabetes-related complications [5]. Additionally, knowledge of micronutrient intake can guide dietary recommendations and lifestyle modifications aimed at improving metabolic control, reducing the risk of complications, and promoting overall health in individuals with diabetes or prediabetes [6]. Research suggests a multifaceted role for vitamin D in insulin sensitivity, with studies showing an inverse relationship between vitamin D deficiency and insulin function [7]. This deficiency may also contribute to a higher risk of complications from diabetes, including coronary heart disease, retinopathy, and neuropathy. Furthermore, adequate levels of antioxidants, particularly vitamins C and E, might be crucial for managing diabetes and its complications [8]. Moreover, micronutrient deficiencies, such as those in selenium, zinc, copper, iron, chromium, and iodine, as well as imbalanced levels of other trace elements, can result in difficulties in regulating blood sugar and insulin resistance [9]. Magnesium deficiency weakens cellular defenses against oxidative damage, making individuals with diabetes more susceptible to its harmful effects. This accelerates the development of diabetes-related complications. Interestingly, lower serum zinc levels were also observed in patients with multiple microvascular complications, suggesting a potential link between zinc deficiency and these complications [9].

Dietary pattern analysis provides a comprehensive understanding of dietary habits and their impact on health outcomes, considering the complex interactions between various dietary factors. However, analyzing micronutrient patterns goes beyond this by revealing the synergistic or antagonistic interactions between different micronutrients [10]. Assessing micronutrient intake against recommended dietary intakes (RDI) and obtaining information on micronutrient patterns contribute to a more holistic picture of micronutrient intake. Few studies have assessed the association between micronutrient patterns and conditions such as sarcopenia [11] or cognitive function [10]. However, research specifically examining the relationship between micronutrient patterns and diabetes or prediabetes remains limited. Moreover, research investigating the intake of specific vitamins or minerals in diabetic patients in Romania is limited. The objectives of the study are 1) to describe the micronutrient intake in patients with prediabetes (PD) and type 2 diabetes (T2DM) compared to RDI and 2) to investigate the associations between micronutrient patterns and anthropometric measurements and biomarkers of glycemic control and lipid profile. This research will contribute to the current understanding of micronutrient intake and dietary habits among individuals with prediabetes and diabetes. Specifically, it focuses on a Romanian population, known for its unique food choices and preferences, thus offering valuable insights into regional variations in dietary patterns.

## Materials and methods

Research design and subjects

This was a cross-sectional study conducted at the Regional Clinical Hospital of Suceava, Diabetology Clinic, on 601 patients, of which 349 with T2DM and 252 with PD, aged between 18 and 75 years old. Individuals with any chronic gastrointestinal disease, significant immunodeficiency, eating disorders, psychiatric diseases, cancer, under systemic antibiotics in the last six weeks or probiotics in the last three months or drugs that can influence weight or metabolism, under insulin treatment or its analogs, pregnancy or breastfeeding, persons with special diets (vegetarian or Atkins) or with substance abuse or excessive intake of alcohol or caffeine or an inability or unwillingness to comply with the protocol were excluded from the study. This study was conducted according to the guidelines stipulated in the Declaration of Helsinki. All procedures were approved by the University of Suceava Research Ethics Committee (protocol number: 11733/14.07.2020) and by the University of Medicine and Pharmacy ‘’Grigore T. Popa‘’, Iasi, Romania. All participants signed a written informed consent before the study commenced.

Dietary assessment and identification of micronutrient patterns

Dietary intake was evaluated using a valid and reliable, 130-item semi-quantitative food frequency questionnaire (FFQ) to determine the usual dietary intake during the year preceding the assessment. The questionnaire has been used in previous studies and was validated for the Romanian population [12]. Nutrient intake was estimated using the FETA-FFQ EPIC Tool for Analysis software [13]. To identify micronutrient patterns, we employed a set of 20 micronutrients, consisting of 10 vitamins and 10 minerals. The dietary intake of these micronutrients was computed per 1,000 kcal of energy intake and used as input variables for analysis. A significant Bartlett's test of sphericity (p < 0.001) and a Kaiser-Meyer-Olkin (KMO) value ≥ 0.50 indicated the statistical correlation between variables and the adequacy of the sample size. Micronutrient patterns were extracted from factor analysis using principal component analysis (PCA) with varimax rotation. The number of micronutrient patterns retained was based on the scree plot and eigenvalues > 2. For each identified micronutrient pattern, vitamins and minerals with factor loadings ≥ 0.35 or ≤ - 0.35 were considered major contributors. Micronutrient patterns were named based on the top 3 micronutrients with the highest factor loading in each pattern. Each participant received a score for each micronutrient pattern, which was then categorized into tertiles for further statistical analysis. Participants in the highest tertile were considered to have the highest adherence to the corresponding micronutrient pattern. In this study, we sourced the DRIs for micronutrients from the Dietary Guidelines for Americans 2015-2020 [14].

Anthropometric measurements

All measurements were performed by a trained dietitian using standardized procedures. Blood pressure was measured with the standardized and calibrated sphygmomanometer (Omron M2) twice in the non-dominant arm after 10 minutes of rest in the sitting position. Blood pressure was determined as the average of the two measurements.

Body weight was measured using a standard scale (Omron HN 288), which was placed on a hard-floor surface, and the participants were weighed wearing light clothing and barefoot. Participants were asked to remove their shoes before their height was measured using a stadiometer, with the measurement reported to the nearest 0.1 cm. The body mass index (BMI) was calculated by dividing the weight in kilograms by the height in meters squared. Body fat (BF) was estimated by the bioelectrical impedance method, using a body analyzer (Omron HBF-306C). Waist circumference (WC) was obtained by inelastic tape measure, with accuracy of 0.1 cm, directly on the skin, at the approximate midpoint between the lower margin of the last palpable rib and the top of the iliac crest.

Biomarker analysis

Blood samples were drawn by trained and experienced nurses through the antecubital arm vein of all study participants, after 12 hours of overnight fasting, and blood samples were analyzed immediately. Fasting plasma glucose (FPG), glycated hemoglobin (HbA1c), serum insulin (SI), total cholesterol (TC), triglycerides (TG), HDL cholesterol (HDL), and LDL cholesterol (LDL) were analyzed by standard laboratory methods using certified assays. The laboratory tests were conducted in a private licensed laboratory.

Data analysis

To evaluate differences between PD and T2DM groups, a t-test was used for continuous variables and χ2 for categorical variables. The relationships between micronutrient patterns, anthropometric measurements, and biomarkers were assessed by using the Pearson correlation coefficient when both variables were normally distributed or the Spearman correlation coefficient when the data did not follow a normal distribution. A one-way ANOVA was used to compare the anthropometric and biomarkers between micronutrient patterns tertiles. One sample t-test was performed to compare the mean micronutrient intakes with the RDI. A p-value < 0.05 was considered statistically significant. Data are presented as mean ± standard error of the mean (SEM) for continuous variables or sum (percentage) for categorical variables. All statistical analyses were performed with IBM SPSS Statistics for Windows (version 28; Armonk, NY).

## Results

Study participants’ characteristics

The general characteristics, anthropometric measurements, and biomarkers of the study participants are presented in Table 1. Compared to patients with PD, patients with T2DM had a higher WC (105.4 ± 0.8 vs. 102.4 ± 0.9 cm; p = 0.010), SI (16.0 ± 0.7 vs. 13.6 ± 0.8 µIU/mL; p < 0.001), and TG (166.9 ± 7.8 vs. 135.5 ± 6.1 mg/dL; p = 0.003). HDL was significantly lower in patients with T2DM compared with those with PD (50.9 ± 0.9 vs. 55.5 ± 1.0 mg/dL; p = 0.001).

**Table 1:** General characteristics, anthropometric measurements, and biomarkers of participants. Categorical variables are presented as sum and percentages, and continuous variables are presented as mean ± SEM. PD, prediabetes; T2DM, type 2 diabetes; SBP, systolic blood pressure; DBP, diastolic blood pressure; WC, waist circumference; BMI, body mass index; BF, body fat; FPG, fasting plasma glucose; HbA1c, glycated hemoglobin; SI, serum insulin; TC, total cholesterol, TG, triglycerides; HDL, high-density lipoprotein; LDL, low-density lipoprotein; a, b significantly different between the two groups for age, WC, FPG, HbA1c, SI, TG and HDL

Variables	PD (n=252)	T2DM (n=349)	p value
Age (years)	59.9 ± 0.8^a^	62.2 ± 0.5^b^	0.017
Men n (%)	87 (34.5%)	131 (37.5%)	0.251
Women n (%)	165 (65.5%)	218 (62.5%)	
Urban n (%)	157 (62.3%)	206 (59.0%)	0.216
Rural n (%)	95 (37.7%)	143 (41.0%)	
SBP (mm Hg)	141.7 ± 1.3	144.4 ± 0.9	0.111
DBP (mm Hg)	85.9 ± 0.7	87.4 ± 0.6	0.118
Weight (kg)	86.3 ± 1.1	87.2 ± 0.8	0.515
WC (cm)	102.4 ± 0.9^a^	105.4 ± 0.8^b^	0.010
BMI (kg/m^2^)	31.5 ± 0.4	31.6 ± 0.3	0.718
BF (%)	35.4 ± 0.5	36.3 ± 0.6	0.281
FPG (mg/dL)	121.4 ± 2.2^a^	167.7 ± 3.3^b^	< 0.001
HbA1c (%)	5.8 ± 0.1^a^	7.9 ± 0.1^b^	0.020
SI (µIU/mL)	13.6 ± 0.8^a^	16.0 ± 0.7^b^	< 0.001
TC (mg/dL)	190.0 ± 2.9	196.5 ± 2.8	0.117
TG (mg/dL)	135.5 ± 6.1^a^	166.9 ± 7.8^b^	0.003
HDL (mg/dL)	55.5 ± 1.0^a^	50.9 ± 0.9^b^	0.001
LDL (mg/dL)	112.7 ± 2.3	116.9 ± 2.0	0.167

Vitamin and mineral intake

T2DM patients had a significantly lower intake of vitamin E (9.4 ± 0.2 vs. 10.1 ± 0.3 mg; p = 0.048), vitamin D (44.3 ± 1.1 vs. 48.9 ± 1.7 IU; p = 0.020), and thiamin (1.3 ± 0.1 vs. 1.4 ± 0.1 mg; p = 0.013) compared to PD patients. Furthermore, T2DM patients showed reduced intake of all micronutrients compared to those with PD, except for copper, where intake levels were comparable between the two groups, although these findings did not reach statistical significance (Table 2).

**Table 2:** Micronutrient intake of participants. Values are mean ± SEM. PD, prediabetes; T2DM, type 2 diabetes; Vit. vitamin; RAE, retinol activity equivalents; AT, alpha-tocopherol; IU, international units; DFE, dietary folate equivalents; Ca, calcium; Mg, magnesium; P, phosphorus; K, potassium; Na, sodium; Zn, zinc; Cu, copper; Mn, manganese; Se, selenium; a, b significantly different between the groups for vitamin E, vitamin D, and thiamin

Micronutrient	PD (n=252)	T2DM (n=349)	p value
Vit. A RAE (mcg)	353.1 ± 14.1	343.0 ± 9.1	0.860
Vit. E AT (mg)	10.1 ± 0.3^a^	9.4 ± 0.2^b^	0.048
Vit. D (IU)	48.9 ± 1.7^a^	44.3 ± 1.1^b^	0.020
Vit. C (mg)	84.5 ± 2.8	83.1 ± 2.5	0.712
Thiamin (mg)	1.4 ± 0.1^a^	1.3 ± 0.1^b^	0.013
Riboflavin (mg)	1.6 ± 0.1	1.5 ± 0.1	0.481
Niacin (mg)	21.1 ± 0.5	20.1 ± 0.4	0.132
Vit. B6 (mg)	1.6 ± 0.1	1.5 ± 0.1	0.073
Vit. B12 (mcg)	5.5 ± 0.2	5.4 ± 0.2	0.711
Folate DFE (mcg)	231.7 ± 5.8	223.3 ± 4.8	0.273
Ca (mg)	750.7 ± 21.8	727.6 ± 17.2	0.401
Iron (mg)	9.3 ± 0.2	8.9 ± 0.1	0.079
Mg (mg)	245.4 ± 5.7	231.1 ± 4.6	0.053
P (mg)	1165.2 ± 26.3	1114.8 ± 21.3	0.134
K (mg)	2850.9 ± 64.2	2714.7 ± 49.8	0.090
Na (mg)	2889.5 ± 68.9	2759.0 ± 54.1	0.132
Zn (mg)	7.9 ± 0.1	7.5 ± 0.1	0.076
Cu (mg)	1.2 ± 0.1	1.2 ± 0.1	0.496
Mn (mg)	2.7 ± 0.1	2.5 ± 0.1	0.064
Se (mcg)	69.8 ± 1.7	66.0 ± 1.4	0.092

Table 3 presents the vitamin intake across gender and age groups in both the PD and T2DM groups, including the vitamin intake as a percentage of the RDI. Among men in the PD group, those aged 31-50 years had a statistically significant lower intake of vitamin A compared to those 51-75 age group (173.9 ± 33.4 vs. 336.5 ± 17.4 mcg; p = 0.002). Conversely, in the T2DM group, men aged 31-50 had significantly higher intakes of vitamin C (117.2 ± 16.3 vs. 81.5 ± 4.6 mg; p = 0.016) and vitamin B6 (1.9 ± 0.1 vs. 1.5 ± 0.1 mg; p = 0.007) than those in the 51-75 age group. Furthermore, in the T2DM group, women aged 31-50 showed significantly higher intake of vitamin C (114.4 ± 11.7 vs. 78.7 ± 3.1 mg; p = 0.002) compared to those aged 51-75. Additionally, men aged 31-50 in the PD group had significantly lower intakes of vitamin A (173.9 ± 33.4 vs. 353.9 ± 28.8 mcg; p = 0.001) and vitamin C (68.7 ± 8.0 vs. 117.2 ± 16.3 mg; p = 0.028) compared to their counterparts in the T2DM group. Similarly, women aged 51-75 in the PD group had significantly higher intakes of vitamin A (397.1 ± 22.4 vs. 339.3 ± 13.0 mcg; p = 0.027) and thiamin (1.3 ± 0.1 vs. 1.2 ± 0.1 mg; p = 0.017) compared to those in the T2DM group.

**Table 3:** The intake of vitamins by age group and the comparison with RDI. Variables are presented as mean ± SEM and as percentages of the RDI. PD, prediabetes; T2DM, type 2 diabetes; Vit. vitamin; RDI, recommended dietary intake; RAE, retinol activity equivalents; AT, alpha-tocopherol; IU, international units; DFE, dietary folate equivalents; a, b represent statistically significant differences between patients of the same sex, but from different age groups; *, & represent statistically significant differences between patients of the same sex and age group

Vitamin	PD patients (n=252)				T2DM patients (n=349)			
	Male 31–50 years	Male 51–75 years	Female 31–50 years	Female 51–75 years	Male 31–50 years	Male 51–75 years	Female 31–50 years	Female 51–75 years
Vit. A RAE (mcg) (% of the RDI)	173.9 ± 33.4^a*^ (19.3%)	336.5 ± 17.4^b^ (37.4%)	322.9 ± 44.8 (46.1%)	397.1 ± 22.4^*^ (56.7%)	353.9 ± 28.8^&^ (39.3%)	350.6 ± 15.6 (39.0%)	337.1 ± 23.9 (48.2%)	339.3 ± 13.0^&^ (48.5%)
Vit. E AT (mg) (% of the RDI)	11.8 ± 2.2 (78.7%)	9.9 ± 0.4 (66.0%)	11.3 ± 1.2 (75.3%)	9.8 ± 0.3 (65.3%)	10.6 ± 0.8 (70.7%)	9.5 ± 0.3 (63.3%)	11.2 ± 0.8 (74.7%)	9.1 ± 0.3 (60.7%)
Vit. D (IU) (% of the RDI)	59.6 ± 12.0 (9.9%)	49.0 ± 2.1 (8.2%)	59.8 ± 7.9 (10.0%)	43.8 ± 1.8 (7.3%)	47.5 ± 3.9 (7.9%)	44.9 ± 1.8 (7.5%)	49.5 ± 6.8 (8.3%)	43.3 ± 1.6 (7.2%)
Vit. C (mg) (% of the RDI)	68.7 ± 8.0^*^ (76.3%)	85.0 ± 4.5 (94.4%)	98.0 ± 12.0 (130.7%)	80.1 ± 3.3 (106.8%)	117.2 ± 16.3^a&^ (130.2%)	81.5 ± 4.6^b^ (90.6%)	114.4 ± 11.7^a^ (152.5%)	78.7 ± 3.1^b^ (104.9%)
Thiamin (mg) (% of the RDI)	1.4 ± 0.1 (116.7%)	1.5 ± 0.1 (125.0%)	1.4 ± 0.1 (127.3%)	1.3 ± 0.1^*^ (118.2%)	1.5 ± 0.1 (125.0%)	1.3 ± 0.1 (108.3%)	1.4 ± 0.1 (127.3%)	1.2 ± 0.1^&^ (109.1%)
Riboflavin (mg) (% of the RDI)	1.4 ± 0.2 (107.7%)	1.5 ± 0.1 (115.4%)	1.5 ± 0.1 (136.4%)	1.5 ± 0.1 (136.4%)	1.9 ± 0.2 (146.2%)	1.5 ± 0.1 (115.4%)	1.5 ± 0.1 (136.4%)	1.4 ± 0.1 (127.3%)
Niacin (mg) (% of the RDI)	18.7 ± 2.0 (116.9%)	21.8 ± 0.7 (136.3%)	22.0 ± 1.9 (157.1%)	20.6 ± 0.6 (147.1%)	22.7 ± 2.0 (141.9%)	20.3 ± 0.6 (126.9%)	21.0 ± 1.2 (150.0%)	19.7 ± 0.5 (140.7%)
Vit. B6 (mg) (% of the RDI)	1.6 ± 0.2 (123.1%)	1.7 ± 0.1 (100.0%)	1.8 ± 0.1 (138.5%)	1.6 ± 0.1 (106.7%)	1.9 ± 0.1^a^ (146.1%)	1.5 ± 0.1^b^ (88.2%)	1.8 ± 0.1 (138.5%)	1.5 ± 0.1 (100.0%)
Vit. B12 (mcg) (% of the RDI)	6.1 ± 1.0 (254.2%)	5.5 ± 0.3 (229.2%)	5.2 ± 0.5 (216.7%)	5.4 ± 0.3 (225.0%)	7.3 ± 1.3 (304.2%)	5.6 ± 0.4 (233.3%)	5.5 ± 0.9 (229.2%)	5.1 ± 0.2 (212.5%)
Folate DFE (mcg) (% of the RDI)	199.5 ± 33.6 (49.9%)	235.4 ± 8.7 58.9%)	245.3 ± 27.4 (61.3%)	230.7 ± 6.9 (57.7%)	294.5 ± 34.1 (73.6%)	224.2 ± 6.3 (56.1%)	255.9 ± 18.6 (64.0%)	214.4 ± 6.9 (53.6%)

In terms of vitamin intake as a percentage of the RDI, we observed that all patients had a statistically significantly lower intake of vitamin A, vitamin D, and folate compared to the RDI. Conversely, there was a significantly higher intake of vitamin B12 compared to the RDI. Additionally, except for men aged between 31 and 50 years in the PD group, all participants had lower intake levels of vitamin E compared to the RDI. On the other hand, the intake of thiamine, riboflavin, and niacin was significantly higher than the RDI for all participants, except for men aged 31-50 with PD. In terms of vitamin C intake, men aged 31-50 with PD had significantly lower intake levels than the RDI. By contrast, women aged 31-50 with T2DM had significantly higher intake levels of vitamin C than the RDI. Concerning vitamin B6 intake, men aged 51-75 with T2DM exhibited significantly lower intake levels compared to the RDI. However, all women with PD and men and women aged 31-50 with T2DM significantly exceeded the RDI.

Table 4 presents the mineral intake across gender and age groups in both the PD and T2DM groups, including the mineral intake as a percentage of the RDI. Among men with T2DM, those aged 31-50 exhibited significantly higher intakes of magnesium (284.5 ± 26.8 vs. 233.3 ± 7.0 mg; p = 0.023), phosphorus (1,366.2 ± 124.1 vs. 1,149.0 ± 34.1 mg; p = 0.045), and potassium (3,433.7 ± 264.0 vs. 2,732.9 ± 76.0 mg; p = 0.004) in contrast to their counterparts aged 51-75. Similarly, within the T2DM group, women aged 31-50 showed significantly higher intakes of magnesium (273.4 ± 17.1 vs. 220.9 ± 6.4 mg; p = 0.034) and potassium (3,153.8 ± 187.7 vs. 2,610.6 ± 69.6 mg; p = 0.043) compared to those aged 51-75. Furthermore, women aged 51-75 in the PD group had significantly higher intakes of calcium (774.5 ± 27.8 vs. 694.2 ± 23.2 mg; p = 0.029), magnesium (241.2 ± 6.4 vs. 220.9 ± 6.4 mg; p = 0.035), phosphorus (1,167.1 ± 30.8 vs. 1,069.5 ± 29.3 mg; p = 0.028), sodium (2,925.5 ± 85.4 vs. 2,659.5 ± 78.7 mg; p = 0.028), zinc (7.8 ± 0.2 vs. 7.1 ± 0.2 mg; p = 0.041), and manganese (2.7 ± 0.1 vs. 2.4 ± 0.1 mg; p = 0.034) compared to their counterparts in the T2DM group.

**Table 4:** The intake of minerals by age group and the comparison with RDI. Variables are presented as mean ± SEM and as percentages of the RDI. PD, prediabetes; T2DM, type 2 diabetes; RDI, recommended dietary intake; AI, adequate intake; UL, tolerable upper intake level; Ca, calcium; Mg, magnesium; P, phosphorus; K, potassium; Na, sodium; Zn, zinc; Cu, copper; Mn, manganese; Se, selenium; a, b represent statistically significant differences between patients of the same sex, but from different age groups; *, & represent statistically significant differences between patients of the same sex and age group

Mineral	PD patients (n=252)				T2DM patients (n=349)			
	Male 31–50 years	Male 51–75 years	Female 31–50 years	Female 51–75 years	Male 31–50 years	Male 51–75 years	Female 31–50 years	Female 51–75 years
Ca (mg) (% of the RDI)	638.6 ± 136.9 (63.9%)	756.1 ± 38.1 (75.6%)	717.0 ± 80.2 (71.7%)	774.5 ± 27.8^*^ (64.5%)	925.8 ± 103.4 (92.6%)	759.7 ± 28.0 (76.0%)	744.9 ± 71.9 (74.5%)	694.2 ± 23.2^&^ (57.9%)
Iron (mg) (% of the RDI)	9.3 ± 1.4 (116.3%)	9.6 ± 0.3 (120.0%)	10.4 ± 1.1 (57.8%)	8.9 ± 0.2 (111.3%)	10.7 ± 1.0 (133.8%)	9.0 ± 0.2 (112.5%)	9.7 ± 0.6 (53.9%)	8.5 ± 0.2 (102.5%)
Mg (mg) (% of the RDI)	242.2 ± 35.4 (57.7%)	248.4 ± 9.4 (59.1%)	259.3 ± 27.0 (81.0%)	241.2 ± 6.4^*^ (73.4%)	284.5 ± 26.8^a^ (67.7%)	233.3 ± 7.0^b^ (55.5%)	273.4 ± 17.1^a^ (85.4%)	220.9 ± 6.4^b&^ (69.0%)
P (mg) (% of the RDI)	1070.2 ± 167.8 (152.9%)	1185.4 ± 40.3 (169.3%)	1171.8 ± 121.8 (167.4%)	1167.1 ± 30.8^*^ (166.7%)	1366.2 ± 124.1^a^ (195.2%)	1149.0 ± 34.1^b^ (164.1%)	1188.9 ± 74.0 (169.8%)	1069.5 ± 29.3^&^ (152.8%)
K (mg) (% of the AI)	2857.6 ± 431.7 (60.8%)	2899.6 ± 115.6 (61.7%)	3024.5 ± 283.3 (64.4%)	2775.7 ± 68.4 (59.1%)	3433.7 ± 264.0^a^ (73.1%)	2732.9 ± 76.0^b^ (58.1%)	3153.8 ± 187.7^a^ (67.1%)	2610.6 ± 69.6^b^ (55.5%)
Na (mg) (% of the UL)	2634.8 ± 392.2 (114.6%)	2995.2 ± 130.3 (130.2%)	2791.5 ± 248.2 (121.4%)	2925.5 ± 85.4^*^ (127.2%)	2998.3 ± 194.1 (130.4%)	2903.4 ± 85.5 (126.2%)	2689.2 ± 111.3 (116.9%)	2659.5 ± 78.7^&^ (115.6%)
Zn (mg) (% of the RDI)	7.8 ± 1.1 (70.9%)	8.1 ± 0.2 (73.6%)	8.1 ± 0.9 (101.3%)	7.8 ± 0.2^*^ (97.5%)	9.1 ± 0.8 (82.7%)	7.8 ± 0.2 (70.9%)	7.9 ± 0.5 (98.8%)	7.1 ± 0.2^&^ (88.8%)
Cu (mg) (% of the RDI)	1.3 ± 0.2 (144.4%)	1.2 ± 0.1 (133.3%)	1.3 ± 0.1 (144.4%)	1.1 ± 0.1 (122.2%)	1.5 ± 0.2 (166.7%)	1.2 ± 0.1 (133.3%)	1.3 ± 0.1 (144.4%)	1.1 ± 0.1 (122.2%)
Mn (mg) (% of the AI)	2.5 ± 0.4 (108.7%)	2.9 ± 0.1 (126.1%)	2.7 ± 0.3 (150.0%)	2.7 ± 0.1^*^ (150.0%)	2.9 ± 0.4 (126.1%)	2.6 ± 0.1 (113.0%)	3.0 ± 0.2 (166.7%)	2.4 ± 0.1^&^ (133.3%)
Se (mcg) (% of the RDI)	65.7 ± 9.6 (119.5%)	76.3 ± 3.0 (138.7%)	67.8 ± 6.6 (123.3%)	67.5 ± 2.0 (122.7%)	80.5 ± 8.2 (146.4%)	68.7 ± 2.4 (124.9%)	62.8 ± 4.6 (114.2%)	63.5 ± 1.8 (115.5%)

Regarding mineral intake as a percentage of the RDI, we observed that all patients had a statistically significantly lower intake of magnesium and potassium compared to the RDI. In contrast, there was a significantly higher intake of copper compared to the RDI. On the other hand, the intake of phosphorus was significantly higher than the RDI for all participants, except for men aged 31-50 with PD. Men aged 31-50 with T2DM were the only subgroup not showing a significantly lower intake of calcium compared to the RDI. Regarding iron intake, significantly lower levels than the RDI was observed in women aged 31-50 from both groups, while significantly higher levels than the RDI were noted in men and women aged 51-75 with PD and in men with T2DM. For zinc intake, significantly lower levels than the RDI were observed in men from both groups and in women aged between 31 and 50 with T2DM. Conversely, significantly higher levels than the RDI were observed in terms of manganese intake in all participants, except for men aged between 31 and 50 in both groups. Similarly, higher intakes than the RDI were observed in all patients for selenium, except for women aged between 31 and 50 in both groups and men aged 31 and 50 with PD, and for sodium, except for women and men aged 31-50 with PD.

Micronutrient patterns

Table 5 presents the factor loading matrix for all analyzed micronutrients, revealing their contributions to the six identified micronutrient patterns. Additionally, the table shows the variance explained by each micronutrient pattern. Using PCA, three micronutrient patterns were identified in the PD group, collectively explaining 55.6% of the variance, and three micronutrient patterns were identified in the T2DM group, collectively explaining 59.8% of the variance. The Iron-Mn-Se pattern included iron, manganese, selenium, magnesium, niacin, zinc, thiamin, and sodium. The Vit.C-K-Folate pattern comprised vitamin C, potassium, folate, vitamin A, vitamin B6, and vitamin E. The Vit.B2-P-Vit.B12 pattern was characterized by positive factor loadings for riboflavin, phosphorus, vitamin B12, calcium, and copper, with a negative factor loading for vitamin E. The K-Folate-Mg pattern consisted of potassium, folate, magnesium, vitamin C, thiamine, vitamin A, vitamin B6, manganese, and sodium, with a negative factor loading for vitamin D. The Iron-Se-Vit.B3 pattern was composed of selenium, iron, niacin, copper, and zinc. The Vit.B2-P-Ca pattern was characterized by positive factor loadings for riboflavin, phosphorus, calcium, vitamin B12, and zinc, with a negative factor loading for vitamin E.

**Table 5:** Factor loading matrix for major micronutrient patterns identified by factor analysis in PD and T2DM patients. Vitamins and minerals with factor loadings  ≥ 0.35 and ≤ − 0.35 are considered significant contributors in each micronutrient pattern. PD, prediabetes; T2DM, type 2 diabetes; Vit. vitamin; Mn, manganese; Se, selenium; K, potassium; Vit. B2, riboflavin; P, phosphorus; Mg, magnesium; Vit. B3, niacin; Ca, calcium.

Nutrient	PD (n=252)			T2DM (n=349)		
	Iron-Mn-Se	Vit.C-K-Folate	Vit.B2-P-Vit.B12	K-Folate-Mg	Iron-Se-Vit.B3	Vit.B2-P-Ca
Iron	0.757	-	-	-	0.731	-
Manganese	0.745	-	-	0.531	-	-
Selenium	0.744	-	-	-	0.739	-
Magnesium	0.651	-	-	0.751	-	-
Niacin	0.641	-	-	-	0.662	-
Zinc	0.630	-	-	-	0.638	0.530
Thiamin	0.616	-	-	0.647	-	-
Sodium	0.357	-	-	0.355	-	-
Vit. C	-	0.814	-	0.750	-	-
Potassium	-	0.803	-	0.829	-	-
Folate	-	0.780	-	0.801	-	-
Vit. A	-	0.595	-	0.585	-	-
Vit. B6	-	0.550	-	0.559	-	-
Vit. E	-	0.547	- 0.417	-	-	- 0.404
Riboflavin	-	-	0.913	-	-	0.940
Phosphorus	-	-	0.810	-	-	0.835
Vit. B12	-	-	0.660	-	-	0.591
Calcium	-	-	0.649	-	-	0.748
Copper	-	-	0.378	-	0.659	-
Vit. D	-	-	-	- 0.458	-	-
Variance Explained (%)	28.2	15.3	12.1	31.6	15.8	12.4

**Figure 1:**
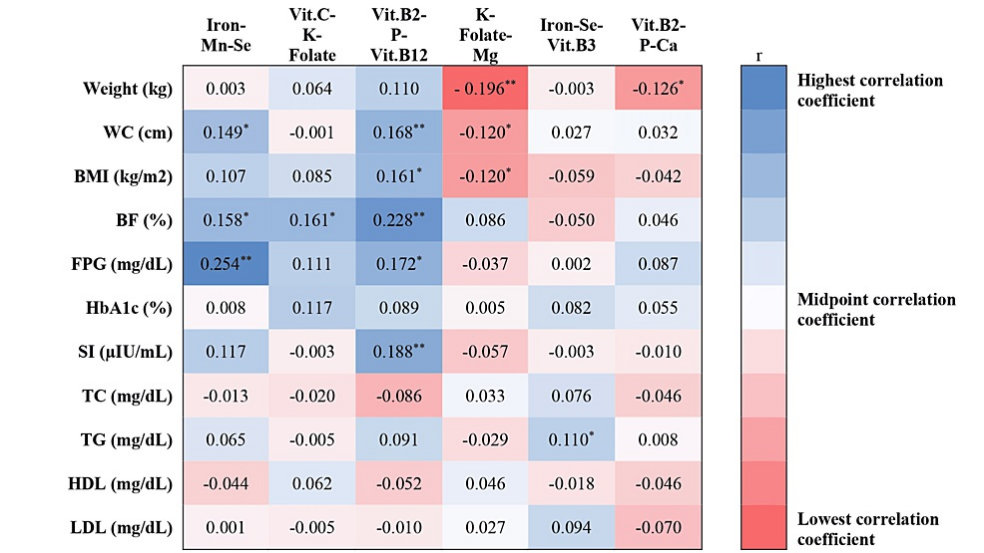
Heat map of the correlation between micronutrient patterns, anthropometric measurements, and biomarkers. Heat map of the correlation between micronutrient patterns, anthropometric measurements, and biomarkers. Blue indicates positive correlations and red indicates negative correlations. The intensity of the color reflects the strength of the correlation. * p < 0.05; ** p < 0.01; r, correlation coefficient; WC, waist circumference; BMI, body mass index; BF, body fat; FPG, fasting plasma glucose; HbA1c, glycated hemoglobin; SI, serum insulin; TC, total cholesterol, TG, triglycerides; HDL, high-density lipoprotein; LDL, low-density lipoprotein; Vit. vitamin; Mn, manganese; Se, selenium; K, potassium; Vit. B2, riboflavin; P, phosphorus; Mg, magnesium; Vit. B3, niacin; Ca, calcium

The correlations between micronutrient patterns, anthropometric measurements, and biomarkers of the participants are depicted in Figure 1. Weight exhibited inverse correlations with K-Folate-Mg (r = -0.196, p < 0.001) and Vit.B2-P-Ca patterns (r = -0.126, p = 0.019). WC demonstrated positive correlations with Iron-Mn-Se (r = 0.149, p = 0.018) and Vit.B2-P-Vit.B12 patterns (r = 0.168, p = 0.008) and an inverse correlation with K-Folate-Mg pattern (r = -0.120, p = 0.025). BMI was positively correlated with Vit.B2-P-Vit.B12 pattern (r = 0.161, p = 0.011) and inversely correlated with K-Folate-Mg pattern (r = -0.120, p = 0.026). BF showed positive correlations with Iron-Mn-Se (r = 0.158, p = 0.013), Vit.C-K-Folate (r = 0.161, p = 0.011), and Vit.B2-P-Vit.B12 patterns (r = 0.228, p < 0.001). FPG was positively correlated with Iron-Mn-Se (r = 0.254, p < 0.001) and Vit.B2-P-Vit.B12 patterns (r = 0.172, p = 0.006), while SI was positively correlated with Vit.B2-P-Vit.B12 pattern (r = 0.188, p = 0.003). TG exhibited a positive correlation with Iron-Se-Vit.B3 pattern (r = 0.110, p = 0.041).

The anthropometric data and biomarkers of PD patients across tertiles of the micronutrient patterns are shown in Table 6. Patients with the lowest adherence to the Iron-Mn-Se pattern had a significantly lower WC compared to those in the third tertile (99.8 ± 1.9 vs. 105.5 ± 1.3 cm; p = 0.044) and a significantly lower FPG compared to those in the second tertile (109.9 ± 3.5 vs. 124.9 ± 4.2 mg/dL; p = 0.005) and third tertile (109.9 ± 3.5 vs. 125.1 ± 3.8 mg/dL; p = 0.003). Regarding the Vit.C-K-Folate pattern, patients with the highest adherence had a significantly higher BF than those in the first tertile (37.1 ± 0.8 vs. 33.5 ± 0.9 %; p = 0.013). Participants with the highest adherence to the Vit.B2-P-Vit.B12 pattern had significantly higher WC (105.8 ± 1.3 vs. 100.4 ± 1.7 cm; p = 0.045) and BF (37.8 ± 0.8 vs. 34.2 ± 0.9 %; p = 0.012) than those in the first tertile, as well as significantly higher FPG (125.6 ± 3.3 vs. 115.2 ± 3.3 mg/dL; p = 0.017) and SI (16.2 ± 1.5 vs. 11.3 ± 0.9 µIU/mL; p = 0.025) compared to those in the second tertile.

**Table 6:** Anthropometric measurements and biomarkers of PD patients across tertiles of micronutrient patterns. Variables are presented as mean ± SEM. PD, prediabetes; T, tertile; WC, waist circumference; BMI, body mass index; BF, body fat; FPG, fasting plasma glucose; HbA1c, glycated hemoglobin; SI, serum insulin; TC, total cholesterol, TG, triglycerides; HDL, high-density lipoprotein; LDL, low-density lipoprotein; Vit. vitamin; Mn, manganese; Se, selenium; K, potassium; Vit. B2, riboflavin; P, phosphorus; *, & represents statistically significant differences between tertiles within the same micronutrient pattern

Variables	Fe-Mn-Se			Vit.C-K-Folate			Vit.B2-P-Vit.B12		
	T1	T2	T3	T1	T2	T3	T1	T2	T3
Weight (kg)	86.7 ± 2.1	85.6 ± 1.7	86.7 ± 1.7	85.1 ± 2.0	87.3 ± 1.9	86.6 ± 1.7	86.2 ± 2.0	83.8 ± 1.9	89.0 ± 1.7
WC (cm)	99.8 ± 1.9^a^	101.9 ± 1.4^ab^	105.5 ± 1.3^b^	103.3 ± 1.7	100.7 ± 1.6	103.2 ± 1.5	100.4 ± 1.7^a^	101.0 ± 1.7^ab^	105.8 ± 1.3^b^
BMI (kg/m^2^)	31.1 ± 0.7	31.2 ± 0.6	32.1 ± 0.7	30.7 ± 0.7	31.9 ± 0.6	31.8 ± 0.6	30.9 ± 0.7	30.8 ± 0.6	32.7 ± 0.6
BF (%)	34.5 ± 1.0	35.5 ± 0.8	36.1 ± 0.9	33.5 ± 0.9^a^	35.5 ± 0.9^ab^	37.1 ± 0.8^b^	34.2 ± 0.9^a^	34.2 ± 0.9^a^	37.8 ± 0.8^b^
FPG (mg/dL)	109.9 ± 3.5^a^	124.9 ± 4.2^b^	125.1 ± 3.8^b^	115.3 ± 3.1	122.8 ± 4.3	124.2 ± 4.2	118.7 ± 4.7^ab^	115.2 ± 3.3^a^	125.6 ± 3.3^b^
HbA1c (%)	5.8 ± 0.1	5.9 ± 0.1	5.8 ± 0.1	5.8 ± 0.0	5.8 ± 0.1	5.9 ± 0.1	5.8 ± 0.1	5.8 ± 0.1	5.9± 0.1
SI (µIU/mL)	12.5 ± 1.0	13.1 ± 1.0	15.1 ± 1.0	14.0 ± 1.5	11.7 ± 0.9	15.0 ± 1.5	13.2 ± 1.5^ab^	11.3 ± 0.9^a^	16.2 ± 1.5^b^
TC (mg/dL)	193.2 ± 4.6	191.9 ± 5.9	184.9 ± 4.7	191.3 ± 4.9	193.4 ± 5.0	185.3 ± 5.3	195.5 ± 4.3	192.5 ± 5.7	182.0 ± 5.0
TG (mg/dL)	146.4 ± 8.2	131.7 ± 7.4	128.5 ± 7.1	137.5 ± 8.7	130.1 ± 9.1	138.9 ± 10.3	140.9 ± 10.5	122.7 ± 7.9	143.0 ± 9.4
HDL (mg/dL)	55.1 ± 1.7	56.2 ± 2.0	55.1 ± 1.7	54.1 ± 1.7	56.6 ± 1.6	55.7 ± 2.0	55.5 ± 1.7	56.7± 2.0	54.2 ± 1.7
LDL (mg/dL)	113.4 ± 3.8	114.7 ± 4.5	110.0 ± 3.6	112.7 ± 3.7	114.5 ± 4.0	110.9 ± 4.2	114.6 ± 3.5	114.5 ± 4.6	108.8 ± 3.8

Patients with T2DM and the highest adherence to the K-folate-Mg pattern had significantly lower weight than those in the first tertile (84.1 ± 1.4 vs. 89.5 ± 1.6 kg; p = 0.025) and significantly lower WC than those in the second tertile (103.2 ± 1.2 vs. 107.7 ± 1.2 cm; p = 0.022). Regarding the Iron-Se-Vit.B3 pattern, patients with the highest adherence had significantly higher LDL than those in the first tertile (120.8 ± 3.4 vs. 109.8 ± 3.0 mg/dL; p = 0.044). Participants with the highest adherence to the Vit.B2-P-Ca pattern had significantly higher FPG than those in the first tertile (174.4 ± 4.8 vs. 155.1 ± 5.1 mg/dL; p = 0.024) (Table 7).

**Table 7:** Anthropometric measurements and biomarkers of T2DM patients across tertiles of micronutrient patterns. Variables are presented as mean ± SEM. T2DM, type 2 diabetes; T, tertile; WC, waist circumference; BMI, body mass index; BF, body fat; FPG, fasting plasma glucose; HbA1c, glycated hemoglobin; SI, serum insulin; TC, total cholesterol, TG, triglycerides; HDL, high-density lipoprotein; LDL, low-density lipoprotein; Vit. vitamin; K, potassium; Mg, magnesium; Se, selenium; Vit. B3, niacin; Vit. B2, riboflavin; P, phosphorus; Ca, calcium; *, & represents statistically significant differences between tertiles within the same micronutrient pattern

Variables	K-Folate-Mg			Iron-Se-Vit.B3			Vit.B2-P-Ca		
	T1	T2	T3	T1	T2	T3	T1	T2	T3
Weight (kg)	89.5 ± 1.6^a^	88.2 ± 1.5^ab^	84.1 ± 1.4^b^	87.7 ± 1.5	87.5 ± 1.5	86.5 ± 1.4	88.8 ± 1.6	88.4 ± 2.6	86.1± 1.1
WC (cm)	106.1 ± 1.2^ab^	107.7 ± 1.2^a^	103.2 ± 1.2^b^	105.3 ± 1.3	106.0 ± 1.3	105.8 ± 1.0	104.7 ± 1.4	106.0 ± 2.3	106.2 ± 0.8
BMI (kg/m^2^)	31.8 ± 0.5	31.9 ± 0.5	31.2 ± 0.5	31.8 ± 0.5	32.0 ± 0.5	31.0 ± 0.4	31.4 ± 0.5	33.0 ± 0.9	31.5 ± 0.4
BF (%)	34.9 ± 0.7	37.1 ± 1.6	37.0 ± 0.8	37.2 ± 1.6	36.2 ± 0.7	35.6 ± 0.9	35.3 ± 0.7	35.5 ± 1.5	37.1± 1.0
FPG (mg/dL)	167.6 ± 6.1	168.8 ± 5.4	166.9 ± 6.0	169.2 ± 6.8	171.5 ± 5.5	162.6 ± 5.1	155.1 ± 5.1^a^	172.2 ± 9.7^ab^	174.4 ± 4.8^b^
HbA1c (%)	7.8 ± 0.1	7.9 ± 0.1	8.0 ± 0.2	7.7 ± 0.1	8.0 ± 0.1	8.0 ± 0.2	7.8 ± 0.1	7.8 ± 0.2	8.0 ± 0.1
SI (µIU/mL)	15.9 ± 1.0	16.8 ± 1.4	15.4 ± 1.3	16.1 ± 1.4	18.1 ± 1.4	13.9 ± 0.7	15.8 ± 1.0	14.8 ± 1.4	16.5 ± 1.1
TC (mg/dL)	198.8 ± 4.6	192.3 ± 4.6	198.3 ± 5.3	187.9 ± 4.5	198.9 ± 5.1	202.5 ± 4.7	197.5 ± 4.9	202.9 ± 7.4	194.5 ± 3.8
TG (mg/dL)	168.9 ± 8.5	159.2 ± 10.2	172.6 ± 12.6	165.6 ± 11.0	158.0 ± 7.4	176.9 ± 10.3	156.6 ± 7.6	148.1 ± 10.4	177.0 ± 13.3
HDL (mg/dL)	51.1 ± 1.6	49.1 ± 1.3	52.6 ± 1.8	50.8 ± 1.3	51.2 ± 1.9	50.9 ± 1.4	51.3 ± 1.5	54.2 ± 2.8	50.1 ± 1.3
LDL (mg/dL)	118.9 ± 3.5	115.2 ± 3.2	116.8 ± 3.9	109.8 ± 3.0^a^	120.2 ± 4.0^ab^	120.8 ± 3.4^b^	118.9 ± 3.8	124.4 ± 6.5	114.3 ± 2.6

## Discussion

In this study, we investigated the micronutrient intake relative to the RDI in a group of individuals with PD and T2DM and identified three distinct micronutrient patterns within each group.

In both groups, the RDI was not met for several micronutrients, including vitamin A, vitamin D, folate, magnesium, and potassium. These micronutrients play a crucial role in the prevention and management of diabetes [8,15]. For example, maintaining adequate serum levels of vitamin A or incorporating sufficient beta-carotene into the diet has been reported to improve insulin resistance [16]. Vitamin D deficiency is not only linked to insulin resistance but is also associated with an increased risk of diabetes complications [8], and a low level of folic acid is associated with the severity of diabetic retinopathy [17]. The deficiency of vitamin D and calcium can negatively influence blood sugar, while supplementation with these nutrients may optimize carbohydrate metabolism [18]. A dietary calcium intake of around 750 mg per day is inversely associated with the risk for T2DM [19]. Low magnesium intake is associated with the development of T2DM and metabolic syndrome [20], while low potassium levels are associated with an increased risk for T2DM [21].

Similar results were seen in a study carried out in Italy, albeit in patients with type 1 diabetes, reporting a low adherence to the recommended intake of vitamin E, vitamin D, folate, calcium, and potassium, both in women and men [22]. Additionally, in the same study, a low adherence to selenium and copper, along with an elevated adherence to the recommended intake of vitamin A was reported [22]. Interestingly, our study showed that participants exceeded the RDI for both copper and selenium but failed to reach the RDI for vitamin A. Similarly, a recent study carried out in South Africa reported an intake lower than 50% of the RDI for calcium, iron, folate, and vitamin D in women and men with T2DM and vitamin A in men with T2DM [23]. Our study demonstrated comparable results, except for iron intake, which surpassed the 50% RDI in our participants. Additionally, a study on diabetes patients from the Philippines found an inadequate intake of folate, calcium, and vitamin A [24]. The diabetic individuals from the Philippines had a low intake of thiamine and riboflavin [24]. These results differ from our study, where our participants’ intake exceeded the RDI for thiamine and riboflavin, but the intake of folate, vitamin A, and calcium is similar to those obtained in our study.

In our study, the intake of vitamin B12 was double compared to the RDI in both groups and all age and sex groups. This high B12 intake could be an important factor in the context of metformin treatment since it could prevent vitamin B12 deficiency associated with metformin [25]. Currently, no recommendations are formulated regarding the maximum recommended intake of vitamin B12, because no adverse effects of excess vitamin B12 from food have been reported in healthy adults. However, considering that the food sources of vitamin B12 were meat, fish, eggs, milk, and dairy products, the intake of animal products in the study may be high. In general, various associations have been reported between increased animal protein intake and increased risk for diabetes [26].

We observed a significant statistical association between adherence to the Iron-Mn-Se pattern, associated with food sources such as meat, poultry, seafood, fish, nuts, and beans [14], and WC and FPG. This relationship mirrors findings from other studies, indicating that BMI correlates strongly with low iron levels, while WC correlates with high iron levels [27]. Elevated levels of iron, whether due to dietary intake or conditions of iron overload, have also been linked to an increased risk of diabetes [28,29]. Moreover, increased dietary selenium intake has been associated with a higher risk of T2DM [30].

In previous studies, manganese has shown an inverse association with diabetes and obesity, particularly in people with low iron intake [31]. However, results from our study differed from those related to the K-Folate-Mg pattern, possibly due to the inhibitory effect of high iron intake on manganese absorption [32]. Another study explored the link between dietary magnesium intake and metabolic syndrome. It revealed that higher magnesium intake was associated with a reduced risk of central obesity, elevated TG, decreased HDL, high blood pressure, and elevated blood glucose [33]. These findings align with our study, where adherence to the K-Folate-Mg pattern is inversely correlated with body weight and WC. Consumption of vegetables and fruits that constitute sources of vitamins C, A, and B6, folate, and potassium has been inversely associated with overweight and obesity [34], findings consistent with those observed within the K-Folate-Mg pattern.

Furthermore, besides the aforementioned nutrients, the Vit.C-K-Folate pattern also encompasses vitamin E, primarily sourced from nuts. Adherence to this pattern has been linked to increased BF, likely attributable to the calorie density of nuts, as suggested by Freisling et al. [35] who expressed concerns regarding excessive nut consumption potentially leading to weight gain and long-term obesity. Other dietary patterns such as the Vit.B2-P-Vit.B12 and Vit.B2-P-Ca patterns share common components, including riboflavin, phosphorus, vitamin B12, and calcium. Adherence to these patterns has been associated with elevated FPG. These nutrients are predominantly sourced from animal-derived foods, which have been reported to increase the risk of diabetes [26]. Moreover, Song et al. [36] found that elevated serum copper levels were associated with higher TC and LDL, findings consistent with those related to the Fe-Se-Vit.B3 pattern.

Several limitations of this study should be considered when interpreting these findings. First, although food frequency questionnaires are widely used and accepted, they capture dietary habits from the preceding year. This represents a significant drawback since participants may encounter challenges recalling specific foods or their frequency of consumption, introducing potential errors in accurate reporting. Second, our results are representative of Suceava County in Romania; therefore, one should exercise caution when generalizing the results to other regions of Romania. Finally, it is important to note that our study was cross-sectional. Therefore, inferring a causal relationship between micronutrient intake and anthropometric measurements should be approached with caution. Insights from this study will be valuable in designing future prospective longitudinal studies focusing on micronutrient intake in patients with prediabetes and diabetes. Future research with larger sample sizes or alternative statistical methods designed to address multiple comparisons could further strengthen the results.

## Conclusions

This study demonstrated that T2DM patients had significantly lower vitamin E, vitamin D, and thiamine intake than PD patients. Both T2DM and PD patients had a significantly lower intake of vitamin A, vitamin D, folate, magnesium, and potassium compared to the RDI. Among the identified micronutrient patterns, only the K-folate-Mg pattern exhibited a significant association with reduced body weight and WC. Notwithstanding, future studies are needed to confirm these findings and to determine specific dietary recommendations to promote micronutrient intake in order to meet the RDIs, that will aid in the prevention and management of diabetes.
